# Comparison of The Therapeutic Effect of Syngeneic, Allogeneic, and Xenogeneic Adipose Tissue-Derived Mesenchymal Stem Cells on Abortion Rates in A Mouse Model

**DOI:** 10.22074/cellj.2019.5954

**Published:** 2018-11-18

**Authors:** Fatemeh Rezaei, Seyed Mohammad Moazzeni

**Affiliations:** Department of Immunology, Faculty of Medical Sciences, Tarbiat Modares University, Tehran, Iran

**Keywords:** Cell Therapy, Mesenchymal Stem Cells, Spontaneous Abortion

## Abstract

**Objective:**

Mesenchymal stem cells (MSCs), due to their immunomodulatory functions, are an ideal candidate for the treatment of immune-related diseases. Recurrent spontaneous abortion (RSA) is one of the most common complications of pregnancy which in many cases is related to the immune system disorders. Our previous study has shown that the abortion rate was decreased following the syngeneic MSCs therapy in abortion-prone mice. In this study, the therapeutic effect of syngeneic, allogeneic, and xenogeneic MSCs was compared in a mouse model of RSA.

**Materials and Methods:**

In this experimental study, MSCs were isolated from adipose tissue (ASCs) of CBA/J and BALB/c mice and human. After characterization, ASCs were injected (IP) at day 4 of gestation to female CBA/J mice following their mating with DBA/2 male mice. In the control group, phosphate-buffered saline (PBS) was injected and CBA/J×BALB/c mating was also used as the normal pregnancy control. On day 14.5 of pregnancy, embryo resorption rate was determined.

**Results:**

The abortion rate significantly decreased following the ASCs therapy from syngeneic (6.31%), allogeneic (6.54%), and xenogeneic group (12.36%) compared to ASCs non-treated group (34.4%). There was no statistical difference between ASCs treated groups, however syngeneic and allogeneic ASCs reduced the abortion rate more efficiently than xenogeneic ASC.

**Conclusion:**

The abortion rate was significantly decreased following the intraperitoneal administration of ASCs from various donated sources in abortion-prone mice. These results indicated that the immunogenicity of allogeneic and xenogeneic ASCs is not a contradictory problem for their therapeutic effects on RSA.

## Introduction

Mesenchymal stem cells (MSCs), due to their ability to secrete various immunomodulatory factors, including prostaglandin E2 (PGE2), transforming growth factor-ß (TGF-ß), interleukin 10 (IL-10), human leukocyte antigen G (HLA-G), inducible nitric oxide synthase (iNOS) and their differentiation potential are an appropriate option for cell- based therapy (1, 2). MSCs have been isolated from different organs including bone marrow, adipose tissue, umbilical cord blood, placenta, muscle, liver, and synovial fluid (3-5). However, adipose tissue could be an ideal source of MSCs, because of its availability and simplicity of established techniques to extract abundant MSCs from this tissue. In addition, various studies have shown that adipose-derived MSCs (ASCs) have strong immunomodulatory properties with no side effects (6-8). ASCs’ immunomodulatory effects are due to the secretion of various growth factors and cytokines, as well as direct cell to cell contact (7).

Recurrent spontaneous abortion (RSA) is one of the most common complications of the pregnancy, with a prevalence of 2-5 percentage among pregnant women. A major fraction of RSA is closely related to the maternal immune system disorders, especially the local immune responses at the feto-maternal interface (9-11). Female CBA/J mice mating to male DBA/2 mice are susceptible to abortion because of numerous immunological disorders and are commonly used as a mouse model of immunologic RSA. The rate of embryo resorption by these mice has been reported to be about 2040%, while in normal mice it is 4-5% (12, 13). Our previous study has shown that autologous ASCs therapy could reduce the abortion rate in abortion-prone mice (14). Since ultimately, animal studies have to be generalized to humans and most studies are based on allogeneic cell therapy because the separation of the autologous MSCs is time-consuming, in this study, we compared the effect of human (xenogeneic), allogeneic and syngeneic ASCs on the reduction of abortion rate in an RSA model.

## Materials and Methods

### Mice and experimental design 

In this experimental study, CBA/J female mice (6-8 weeks), BALB/c, and DBA/2 male mice (9-11 weeks) were purchased from Pasteur Institute of Iran (Tehran, Iran). All animals were kept under controlled conditions of temperature, humidity, and light (cycles of 12 hours dark/light). All experimental procedures on animals were followed according to the rules of the Ethical Committee of the Faculty of Medical Science, Tarbiat Modares University IR.TMU.REC.1394.255). CBA/J female mice were mated to either DBA/2 or BALB/c males overnight. Detection of the vaginal plug was considered the day 0.5 of gestation. It is accepted that CBA/J female mating to DBA/2 males mice show immunological abortion and are defined as abortion- prone pregnant mice. The mating of CBA/J mice to BALB/c results in normal pregnancy and is considered as normal pregnant mice in this experiments (15).

Some pregnant mice in the abortion-prone group (CBA/ J×DBA/2) received 10^6^ syngeneic, allogeneic or xenogeneicASCs in phosphate-buffered saline (PBS) intraperitoneally onthe day 4.5 of gestation (implantation window) (ASCs treatedgroup, n=5 for each kind of ASC). Some mice in the samemating pairs received an i.p. injection of PBS as a controlgroup (n=5). CBA/J×BALB/c mating as the normal pregnancycontrol also received PBS (n=5). Animals were sacrificed bycervical dislocation on the day 14.5 of gestation. Afterward, uteri horns were isolated from pregnant mice and the totalnumber of embryo resorption was counted. The percentage ofresorption in experimental groups was calculated accordingto the formula: resorption rate %= (number of resorbed fetus/ number of the total fetus)×100 (16).

### Isolation of mesenchymal stem cells from the adipose tissue 

MSCs were isolated from the abdominal fat of CBA/Jand BALB/c mice (3-5 week), adipose tissue was cut intosmall pieces and digested with 1 mg/mL collagenase type I(Sigma-Alderich, USA) for 30 minutes at 37°C with every10 minutes shaking to get a single cell suspension. Afterneutralization of collagenase with Dulbecco’s Modified Eagle Medium (DMEM, Gibco, UK) containing fetal calfserum (FCS), the separated cells were centrifuged (1500 gfor 15 minutes) and the cell pellet was cultured in DMEMcontaining 10% FCS and kept in 5% CO_2_ at 37°C. After 24 hours, the medium was changed to remove the non-adherentcells. When approximately 70-80% confluence was achieved, the adherent cells were trypsinized and harvested. Passage 2cells were used for injection. 

Human ASCs were isolated from Lipoaspirate samplesas described by Zhu et al. (17). In brief, adipose tissue wasobtained after liposuction with informed consent and digestedby collagenase type I (1 mg/mL, Sigma-Alderich, USA). Forthis purpose, adipose samples were mixed with collagenasesolution and placed at 37°C for 30 minutes. DMEM with10% FCS was used to neutralize collagenase (5 minutes atroom temperature). Then the cell pellets were collected bycentrifugation (1200 g for 10 minutes) and cultured in DMEMcontaining 10% FCS and kept in 5% CO_2_ at 37°C. After removal of non-adherent cells and getting a confluent culture, the cells from the second passage were used for experiments. It has been shown that MSCs lost their stem cells propertiesand enter the senescence during *in vitro* cultures (18). So we chose the second passage for the cell therapy.

### Immunophenotyping of adipose tissue-derived mesenchymal stem cells

The expression of surface markers on MSCs was investigated using the following antibodies. Anti-mouse CD34 (PE, eBiosience, USA), anti-mouse CD44 (APC, BD, USA), anti-mouse CD45 (APC-cy7, Biolegend, USA), anti-mouse CD73 (PE, BD, USA), anti-mouse CD90 (APC, BD, USA), anti-mouse CD105 (PE, eBiosience, USA), anti-mouse Sca1 (FITC, Biolegend, USA), anti-mouse CD3 (PE, BD, USA), anti-human CD90 (APC, Biolegend, USA), anti-human CD105 (APC, Biolegend, USA), anti-human CD29 (PE, eBiosience, USA), anti-human CD45 (FITC, Biolegend, USA) and anti-human CD34 (PE, eBiosience, USA). Passage 2 cells were used for the analysis of cell surface markers by flow cytometry (FACS calibur, Becton Dickinson, USA). For flow cytometry analysis, 10,000 events were counted and data were analyzed using the flowJo software. 

### Multi-lineage differentiation of adipose tissue-derived mesenchymal stem cells

Isolated MSCs from the adipose tissue were cultured in DMEM containing 10% FCS, dexamethasone (0.5 mM, Sigma-Alderich, USA), indomethacin (50 mM, Sigma- Alderich, USA), insulin (5 µM, Sigma-Alderich, USA), and isobuthylmethylxanthine (0.5 mM, Sigma-Alderich, USA) for 3 weeks to induce adipose differentiation. Differentiated cells were assessed using oil red O for adipocyte detection.

To induce the differentiation toward osteocytes, MSCs were incubated in condition medium (DMEM+10% fetal bovine serum) supplemented with ascorbic acid (50 mg/ ml), ß-glycerolphosphate (10 mM), and dexamethasone (0.1 µM). After 3 weeks incubation at 37°C the cells were fixed by formalin 10%, then the cells were stained with Alizarin red (Sigma-Alderich, USA) to detect mineralized matrix of the bone (17, 19).

### Statistical analysis

Statistical analysis of the data was performed using the SPSS version 23 software (IBM company, USA). The differences in resorption rate between experimental groups were analyzed by chi-square (.2) and Fisher’s exact test where appropriate. Data are presented as mean ± SD. The P<0.05 were considered statistically significant. 

## Results

### Isolation of mesenchymal stem cells from adipose tissue and their characterization 

**Fig.1:**
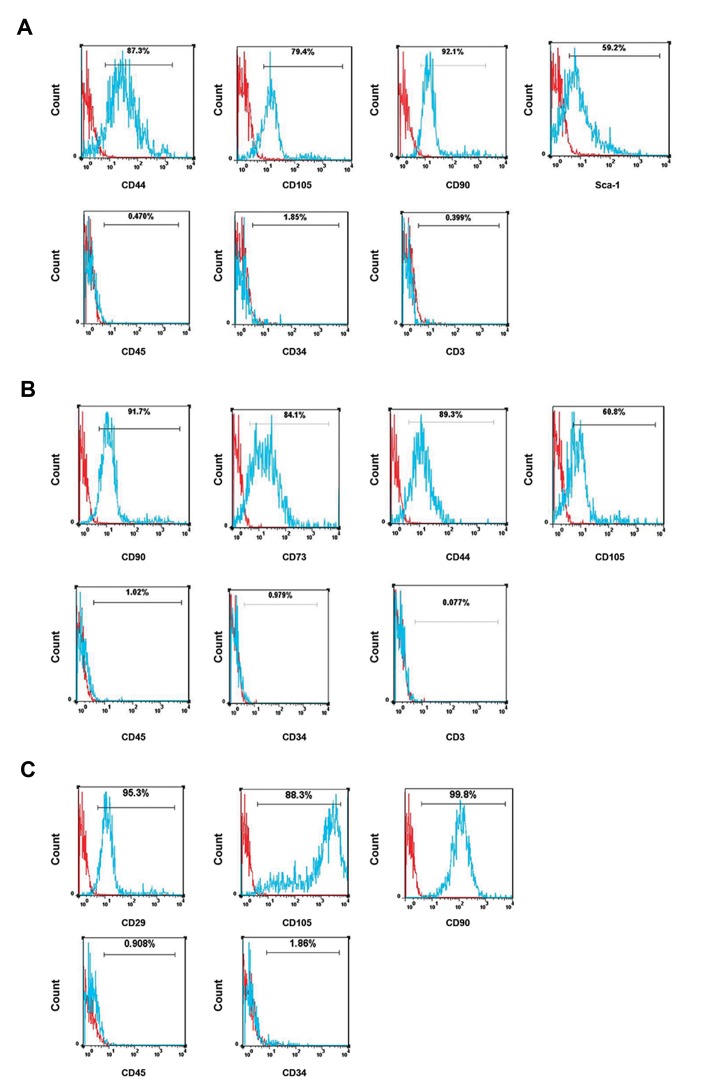
Cell surface phenotype analysis of adipose tissue-derived mesenchymal stem cells (ASCs). **A**. The syngeneic (obtained from CBA/J), **B**. Allogeneic (obtained from BALB/c), and **C**. Xenogeneic ASCs (obtained from human) were analyzed for the expression of cell surface markers at the second passage of cultured cells by flow cytometry. The cells were positive for stem cell markers and negative for the hematopoietic markers in all kinds of ASCs. Histograms show the expression of surface markers (blue) which were plotted against the unstained control (red).

MSCs were isolated from different sources including abdominal fat of CBA/J and BALB/c mice, and human lipoaspirate. Cultured ASCs were fibroblast-like, plastic adherent, and spindle-shaped which were consistent with MSC morphology. Immunophenotyping analysis demonstrated that MSCs cultures from passage 2 in mice were positive for CD105, CD44, Sca-1, CD73, and CD90 and negative for CD45, CD3, and CD34 (Fig .1A, B). Immunophenotyping analysis also demonstrated that isolated MSCs from human liposuction were positive for CD90, CD105, and CD29 and negative for CD45 and CD34 (Fig .1C).

### Differentiation of adipose tissue-derived mesenchymal stem cells into osteogenic and adipogenic lineages

**Fig.2:**
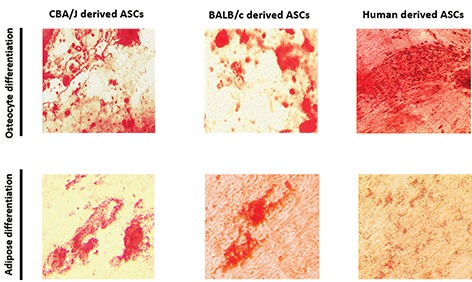
Differentiation potential of adipose tissue-derived mesenchymal stem cells (ASCs). **A**. Osteogenic capability of cells was determined by Alizarin Red staining after 21 days of induction in osteogenic medium and **B**. The ability of ASCs to differentiate into adipocyte was characterized by oil red O staining after being cultured in the adipogeneic medium (scale bars: 50 µm).

To evaluate the multi-lineage differentiation ability of the isolated ASCs, the cells were induced to osteoblast and adipocyte under appropriate culture conditions. In the osteogenic medium, both human and mice ASCs formed the calcium mineralization confirmed by Alizarin red staining (Fig .2A). The ASCs were also cultured in adipogenic medium and revealed that ASCs of all sources formed lipid droplet confirmed by oil red O staining (Fig .2B)

### Adipose tissue-derived mesenchymal stem cells reduced the abortion rate in the abortion-prone mice

**Fig.3:**
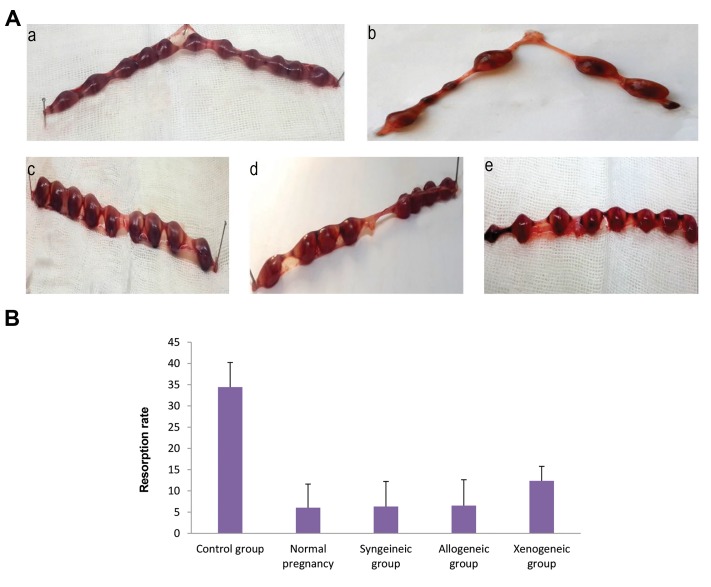
Effect of cell therapy on the embryo resorption rate. Mice wereinjected i.p with syngeneic, allogeneic, and xenogeneic adipose tissue- derived mesenchymal stem cells (ASCs) or PBS on day 4.5 of gestation. On day 14.5 of gestation uteri were removed and investigated for resorption rate. **A**. Representative photographs indicating the implantation sites in normal pregnancy (a), control group (b), syngeneic group (c), allogeneic group (d), and xenogeneic group (e) and **B**. Resorption rate in studied groups. In the ASCs treated groups, the percentage of embryo loss was significantly lower than the control group. The data are presented as mean ± SD.

We observed that the administration of ASCs from all sources at the day 4.5 of pregnancy to CBA/J pregnant mice in CBA/J×DBA/2 matting, significantly reduced the abortion rate compared to the untreated control group which received PBS (P<0.05). The difference in abortion rate down-regulation between syngeneic, allogeneic, and xenogeneic ASCs was not statistically significant, however syngeneic and allogeneic ASCs reduced the abortion rate more efficiently (P=0.0007) than xenogeneic ASC (P=0.014). The percentage of abortion rate on the day 14.5 of gestation in non-treated control group was 34.4% (16 out of 46 implanted fetuses; n=5) in syngeneic ASCstreated group was 6.31% (3 out of 48 implanted fetuses, n=5), in allogeneic ASCs-treated group was 6.54% (3 of 47 implanted fetuses, n=5) in xenogeneic ASCs-treated group was 12.36% (6 of 48 implanted fetuses, n=5). The resorption rate in normal pregnancy group was 6.04% (3 of 49 implanted fetuses, n=5) (Fig .3). 

## Discussion

Our previous studies showed that syngeneic ASCs therapy could reduce the abortion rate in abortion-prone mice and might be considered a promising treatment route for immune-mediated pregnancy loss (14, 20). Here, we showed that allogeneic and xenogeneic ASCs therapy could also reduce the abortion rate in this model. 

Several studies demonstrated that disorders of immune responses play a crucial role in the pathophysiology of RSA (12, 21), so immunomodulatory therapy could be an attractive and hopeful treatment for this disorder. Various sources of MSCs including allogeneic, syngeneic, and xenogeneic have been used in the treatment of different regenerative and auto-immune disorders (22-24). We have used autologous ASCs in our previously mentioned studies to reduce the abortion rate. Because autologous ASCs are not always simply available, in this study, we compared the therapeutic effects of syngeneic, allogeneic, and xenogeneic ASCs in the cell therapy of recurrent pregnancy loss using an appropriate RSA animal model.

Our results showed that the abortion rate was decreased following ASCs therapy in all studied groups. ASCs therapy could significantly reduce the abortion rate from 34.4% in non-treated abortion-prone mice to 6.3%, 6.54%, and 12.36% in syngeneic, allogeneic and xenogeneic ASCs- treated groups, respectively. As seen, all kinds of ASCs remarkably reduced the abortion rate in comparison to the untreated control group.

It has been shown that adverse immune response plays a crucial role in most cases of spontaneous abortion. Dysregulated activities of natural killer cells, T cells, and macrophages, as well as the decreased density of regulatory T cells and altered activities of dendritic cells, are reported to be involved in the etiology of RSA by many investigators (12, 13, 21, 25). Regarding to the aberrant immune response as the main player in most cases of spontaneous abortion and accepted immunomodulatory properties of MSCs (26-28), it could be concluded that downregulation of abortion rate could be mainly due to the immunomodulatory effects of MSCs, which could abrogate or regulate the undesirable immune reactions. Besides, the immunoregulation through a direct cell to cell contact, the most important immunomodulatory factors of MSCs are PGE2, hepatocyte growth factors (HGF), Indoleamine 2, 3-dioxygenase (IDO), nitric oxide (NO), IL-10, and TGF-ß1 which lead to the suppression of B, T, and NK-cell proliferation and DC maturation. MSCs are also reported as strong inducers of regulatory T cells and M2 macrophages (2, 28-33). Several studies also indicated the protection of fetus from abortion through immunosuppressive molecules such as TGF-ß and IL-10 (34, 35). These results suggest that MSCs may improve the pregnancy outcome through the modulation of the adverse immune responses at the feto-maternal interface. 

In this study, we observed no statistical difference among therapeutic effect of the different sources of ASCs, however xenogeneic ASCs had less efficiency compared to syngeneic and allogeneic ASCs. It is likely that the crosstalk between mouse-derived MSCs and mouse immune cells in this model is more effective than xenogeneic (human-derived) MSCs. However, some molecules that induce immunomodulatory function of MSCs are common among species such as PGE2, IL-10, hemeoxygenase-1 (HO-1), and IL-6, but there are some structural differences between these MSCs-derived secretory components between mouse and human which cause a lower response of target cells from the mouse immune system to human- derived cytokines. Direct cell-cell interaction is another mechanism for immunomodulating by MSCs. This interaction is exerted through the cell surface ligands and ligates such as programmed cell death 1 ligand 1 (PDL1), PD1, intercellular adhesion molecule-1 (ICAM-1), vascular cell adhesion molecule-1 (VCAM-1), integrin alpha-4 (ITGA4), and galectin (36, 37). In this case, the structural differences in surface molecules among species do not let an effective cross-talk between the human and mouse cells. MSCs from various species also exert their effects through different mechanisms. Some studies showed that murine MSCs use inducible nitric oxide synthase, while the human MSCs use IDO as a tool for their immunomodulatory properties (30, 38). This could be another possible explanation for the difference in their therapeutic effects in our model.

Although most *in vitro* studies have indicated the immunosuppressive effect of MSCs, several studies have also shown the immunogenicity of these cells for non-syngeneic species. After systemic injection of allogeneic and xenogeneic MSCs, their presence in recipient tissues is probably limited because of the immunological process (29, 31, 38). The effects of syngeneic versus allogeneic MSCs were investigated in EAE and have shown that allogeneic MSCs stimulate the immune responses compared to syngeneic MSCs. However, both treatments had similar curative effects (38). We also observed the same results and there was no difference between therapeutic effects of syngeneic and allogeneic groups. This finding may be related to weak immunogenicity of allogeneic MSCs compared to other cell types from allogenic source, which causes their slow rejection and longer presence in recipient animals (29). For xenogeneic MSCs the immunogenicity could be stronger and more limitary. So the structural differences in implicated molecules in immunomodulation and weak immunogenicity of xenogeneic ASCs could be considered the main reasons for the lower efficiency of these cells in the reduction of the abortion rate.

## Conclusion

The results of the present study demonstrated that, in spite of the weak immunogenicity of allogeneic MSCs, it can be used instead of autologous MSCs. The separation of autologous MSCs is time-consuming and not suitable for the acute conditions. Additionally, MSCs from various donors are somewhat different in their therapeutic effects but allogeneic MSCs can be harvested from the healthy donors and their therapeutic and immunomodulatory efficacy could be investigated for the banking purposes. 
